# Proximity to Water Sources and Avoidance of Human Settlements Drive Wild Boar (*Sus scrofa*) Occupancy During Spring in an Urban-Proximate National Park in South Korea

**DOI:** 10.3390/ani15233423

**Published:** 2025-11-27

**Authors:** Sangjin Lim, Maniram Banjade, Segang Park, Eui-Kyeong Kim, Yungchul Park

**Affiliations:** 1 College of Forest and Environmental Sciences, Kangwon National University, Chuncheon 24341, Republic of Korea; sangjin@kangwon.ac.kr; 2 Wildlife Ecology Institute, ECOEM Inc., Wonju 26465, Republic of Korea; mani88zoo@gmail.com (M.B.); 2000888@naver.com (S.P.); 3 Division of Ecological Survey, Research Institute of Korea National Park, Wonju 26441, Republic of Korea

**Keywords:** Bukhansan National Park, camera trapping, management, occupancy, spring season, wild boar

## Abstract

The wild boar (*Sus scrofa*) is a highly adaptable species that has spread into urban-proximate areas, consequently intensifying human–wildlife conflicts in South Korea. Understanding the range and environmental preferences of this species is crucial for efficient population management. Therefore, we investigated wild boar occupancy in Bukhansan National Park (BNP), a protected area near Seoul. We deployed camera traps at 24 locations from March to May 2022 to investigate spring season habitat use patterns. We used single-season, single-species occupancy modeling to explore the impact of environmental and anthropogenic factors on the distribution of wild boar. During 2208 trap nights, we recorded wild boars at 14 sites, with an average occupancy probability (Ψ) of 0.67 ± 0.03. The distance to human settlements was the best predictor of occupancy, with wild boars avoiding regions near human activity sites. In contrast, proximity to puddles significantly increased detection and occupancy probabilities, indicating the importance of water sources for drinking and wallowing. Wild boars also showed a preference for areas near agricultural lands but exhibited behavioral avoidance of direct human presence. Our spring season findings suggest the need for targeted management strategies that prioritize population control in areas far from settlements but adjacent to water and agricultural boundaries. This study provides critical insights into the spatial ecology of wild boars in urban-proximate landscapes and provides science-based measures for mitigating conflicts and disease risks. We recommend long-term monitoring for the assessment of seasonal variations and efficacy of management interventions.

## Simple Summary

Wild boars (*Sus scrofa*) are increasingly spreading into urban-proximate areas in South Korea, leading to conflicts with humans and agriculture. Through camera traps and occupancy modeling in Bukhansan National Park (BNP) during spring (March–May), we determined key factors driving their distribution. The results show that wild boars avoid areas in close proximity to human settlement; yet they show significant associations with water, such as puddles. They also inhabit habitats near agricultural lands while actively trying to reduce direct encounters with humans. The spring season findings provide key guidance for targeted management strategies aimed at reducing conflicts, crop depredation, as well as disease transmission risks in landscapes characterized by human dominance.

## 1. Introduction

The wild boar (*Sus scrofa*) is one of the most widespread and adaptable mammals in Europe, Asia, and parts of North Africa [1,2]. This generalist species lives in varying landscapes, including forests, grasslands, wetlands, and agricultural areas [3,4]. Wild boars alter soil composition, seed dispersal, and trophic interactions in ecosystems [5]. However, their high reproductive rates, opportunistic feeding, and adaptability have raised ecological and economic concerns [6,7]. Wild boars are often considered agricultural pests because they damage crops, orchards, and natural vegetation by foraging and rooting [8]. Wild boars are also reservoirs for zoonotic diseases, such as African swine fever (ASF), classical swine fever, and foot-and-mouth disease [9,10,11]. Thus, these mammals pose a risk to livestock and human health [11]. Climate change, habitat modification, omnivorous behavior, and the lack of natural predators in some areas have led to population growth, consequently intensifying human–wildlife conflict and requiring integrated management [12]. 

In South Korea, the wild boar population has significantly increased over the past few decades [13] due to a lack of natural predators, such as tigers and leopards, which were extirpated during the early to mid-20th century. Additionally, land-use changes, including agricultural expansion and forest regeneration, have created favorable foraging habitats [13,14]. Once confined to forested and mountainous areas, wild boars are now frequently found in lowland agricultural areas and urban outskirts. They damage crops and infrastructure in these areas; additionally, they pose safety concerns to local communities [15]. The Ministry of Environment (MOE) reported huge economic losses due to wild boar activity, with crop damage costs of billions of Korean Won per year [13]. Wild boars in South Korea have also been linked to ASF, with thousands of cases recorded [16,17,18]. They are also hosts for other zoonotic diseases; therefore, their habitat preferences must be investigated to prevent disease transmission [19].

The control of wild boar populations is challenging because of their adaptability, nocturnal behavior, and rapid reproduction. Government and non-governmental organizations have implemented various management strategies, including regulated culling, trapping, and relocation [4]. However, wild boar populations remain increased despite these efforts [20]. Therefore, more effective science-based management is required. The effective management of wild boar populations requires a comprehensive understanding of their spatial ecology and habitat use. Although wild boar behavior, genetics, diet, activity patterns, and habitat preferences have been extensively studied [4,21,22,23], the environmental and anthropogenic factors underlying their occupancy dynamics remain unclear. Identifying critical habitats and understanding how wild boar interact with them can aid in the prioritization of management and monitoring activities. It can enable governments and conservationists to undertake targeted control measures in high-occupancy areas, thereby improving population management efficiency. Effective methods for the prediction of wild boar distribution and habitat preferences must be developed to facilitate the adaptation of management policies, consequently reducing the risk of continued population growth and conflict with human activities.

Occupancy analysis is a reliable and commonly used method for studying species interactions with ecological and anthropogenic variables [24,25]. This method estimates species distribution based on the presence or absence of species in certain locations and provides crucial information regarding habitat quality and suitability [26,27]. It is especially useful for the study of wildlife in human-dominated areas, as it elucidates species-environment relationships and provides strategies for effective species management [25]. However, the use of occupancy modeling to study wild boar populations in urban-proximate national parks is limited. Therefore, the mechanisms through which environmental and anthropogenic factors influence their distribution and habitat usage remain unclear.

In this study, we aimed to investigate the spring season occupancy of wild boars in Bukhansan National Park (BNP), South Korea, using camera trap-based occupancy modeling. BNP is surrounded by rapidly urbanizing areas and represents a unique setting where human–wildlife interactions are intensifying owing to the growing wild boar population. Specifically, we aimed to identify the critical habitats and factors influencing wild boar presence, with a focus on both environmental and anthropogenic variables. We hypothesized that the occupancy probability would be higher near water sources, such as puddles and rivers, given their reliance on water for hydration and wallowing [28]. Our findings elucidate the factors driving wild boar distribution in urban-proximate landscapes, which will contribute to the development of evidence-based management strategies aimed at mitigating human–wildlife conflicts. Furthermore, our findings will facilitate the effective management of wild boar populations while minimizing conflicts with human activities in the highly urbanized landscape surrounding the BNP.

## 2. Materials and Methods

### 2.1. Study Area

**Figure 1:**
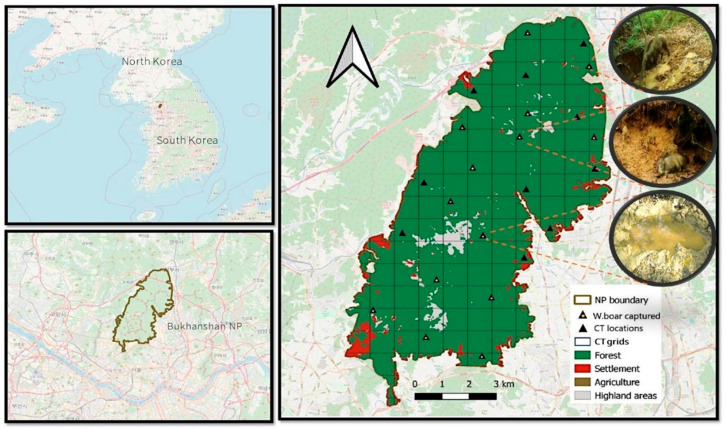
Map of the study area and camera trap (CT) locations within Bukhansan National Park, South Korea. The images on the right (within the circle) depict puddles used for wallowing by wild boars at various CT locations. The white arrow indicates the north.

This study was conducted in BNP, situated in the northeastern region of South Korea (126.9347°–127.0511° E and 37.4314°–37.7317° N) (Figure 1). The park encompasses a core area of 76.92 km^2^ and is located next to Seoul, the country’s capital. It is one of the most frequently visited national parks in South Korea, attracting approximately 10 million visitors annually [29]. Additionally, the park has an average annual temperature of 18.0 °C and receives an annual precipitation of 1291.7 mm, which is slightly higher than the national average (1200 mm). The primary vegetation types in this park include Mongolian oak (*Quercus mongolica*) and Japanese red pine (*Pinus densiflora*) communities that cover approximately 50.33% of the area, followed by mixed forest [30]. The BNP is home to a diverse range of species, including water deer (*Hydropotes inermis*), wild boar (*Sus scrofa*), and other small animals such as the racoon dog (*Nyctereutes procyonoide*), Asian badger (*Meles leucurus*), and Siberian chipmunks (*Eutamias sibiricus*) [31]. In addition, this park is home to various bird species, making it popular for bird watching. 

### 2.2. Sampling Design and Data Collection

We established a grid of 1 × 1 km^2^ cells across the BNP range using QGIS 3.16 (QGIS Development Team, 2014), covering a total area of 76.92 km^2^ (Figure 1). From this grid, 24 cells (approximately 32% of the overall study area) were selected for camera trapping based on their accessibility and suitability for detecting the presence of wild boars. This sampling density was considered sufficient because the selected cells evenly represented the range of environmental conditions and habitat types within BNP, including varying elevations, forest types, and proximity to anthropogenic features. Each of the selected grid cells was equipped with a remote camera (Moultrie’s M-990i, PRADCO Outdoor Brands, Birmingham, AL, USA; and SPYPOINT Force; Skypoint, Surfers Paradise, Australia), systematically positioned 60–100 cm above the ground along animal trails, near rooting signs, and areas with frequent track and scat evidence. This systematic but habitat-informed placement helped minimize detection bias by ensuring that camera traps were deployed in areas of potential wild boar activity across different habitat contexts, not only in the most active zones. Camera traps were installed from March to May 2022. Each camera was active for 92 d, with each 1-month period considered a separate sampling occasion, totaling three sampling occasions during the study period. The shorter time frame was chosen to maintain the demographic and geographic closure required for a single-season occupancy model. Wild boar detection (photographs) and non-detection (lack of photographs) were systematically recorded and compiled for analysis. The detection data obtained from camera traps were used to model site occupancy (ψ), representing the probability that wild boar used a given grid cell during the study period, rather than the intensity of area penetration or movement frequency. The study was conducted in spring, a season with increasing food resources that are likely to influence wildlife movement and habitat utilization [32]. This period was selected to increase detection probability while minimizing seasonal variation in occupancy estimations.

### 2.3. Covariate Selection

We identified the significant biological and anthropogenic factors affecting the distribution of wild boar in BNP based on a comprehensive literature review. Wild boars are extremely adaptable, although their spatial patterns are influenced by habitat characteristics, resource availability, and human activity [13,33,34]. To determine occupancy, we selected eight ecologically relevant covariates: terrain ruggedness index (TRI); normalized difference vegetation index (NDVI); distance to settlements, farmland, roads, rivers, and puddles; as well as forest type (Supplementary Table S1). These factors were selected because of their impact on wild boar foraging behavior, mobility patterns, and responses to human disturbance [33,35,36]. The TRI was included because wild boars prefer diverse environments that offer both shelter and movement corridors. This index was computed using 30 m resolution Shuttle Radar Topographic Mission Digital Elevation Model data (https://srtm.csi.cgiar.org/, accessed on 9 August 2024). The NDVI, a proxy for vegetation production and food availability, was computed using near-infrared and visible red spectral reflectance from Landsat 8 imagery (April 2022), as described by Wu et al. [37]. QGIS was used to determine the distance between settlements, farms, and roadways, as human-modified landscapes influence wild boar movement and habitat selection. Additionally, proximity to water sources such as rivers and puddles was considered because of their roles in hydration and wallowing. Forest species, such as *Quercus mongolica*, *Pinus densiflora*, and mixed forests, were also assessed based on their ability to provide fodder and cover [13,35]. Forest type classification was based on existing vegetation mapping of Oh et al. [30] using a 60% species dominance threshold.

### 2.4. Statistical Analysis

We initially performed correlation analyses for the continuous variables using a threshold of |r| > 0.70 threshold [38]. We included all variables in the analysis as none were substantially associated. We used a single-species, single-season occupancy model to evaluate wild boar habitat usage. This model allows for imperfect detection and uses presence–absence data to provide robust estimates of probability of use (ψ) [24,26]. We utilized an extension of the standard occupancy model [24] that allows for spatially correlated replicates [39], and analyses were performed in R using the “unmarked” package (version 1.5.0) [40,41]. The presence or absence of wild boars was recorded using 1 × 1 km^2^ grids, with 1 indicating detection and 0 indicating non-detection. We used a two-step modeling approach. First, we modeled covariates on detection probability “*p*”, where the parameter was either assumed to be constant or allowed to change as covariates while ψ remained constant in a general model [26]. The previously identified best detection model was fixed, and all possible combinations of site use covariates were varied to model site use probability (ψ). Continuous covariates were standardized on a z-scale, and all covariates were tested for collinearity using Pearson’s correlation test. Covariates with r > 0.6 were excluded from the same model [42]. Furthermore, the models were rated based on their Akaike information criterion (AIC), corrected for small sample sizes [26]. Models with a ΔAIC < 2 were considered well-supported. Models that did not achieve numerical convergence were excluded. 

## 3. Results

### 3.1. Wild Boar Detection and Occupancy Estimates

In total, 2208 trap nights were conducted at 24 survey stations, resulting in the detection of wild boars at 14 stations. The naive occupancy estimate was 0.58, indicating the presence of wild boars in approximately 58% of the surveyed locations. We fitted 30 a priori alternative model combinations (12 for detection and 18 for occupancy) to estimate the wild boar occupancy and detection probabilities. The model-averaged occupancy estimate for wild boars was 0.67 (Standard Error (SE) = 0.03), suggesting a higher probability of occupancy when accounting for detection uncertainty.

### 3.2. Factors Affecting Detection Probability

**Figure 2:**
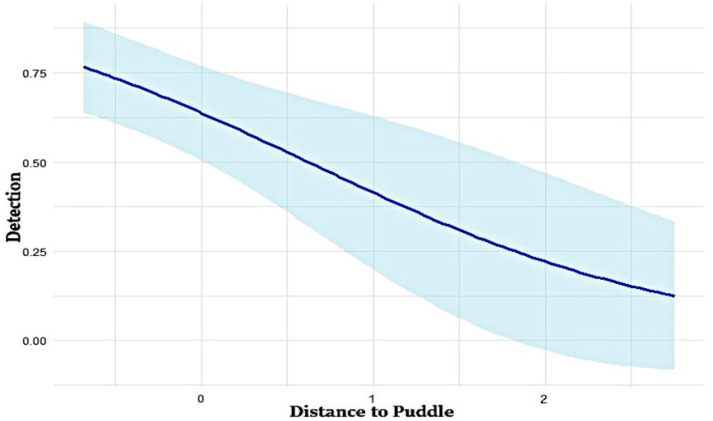
Relationship between distance to puddles and detection probability in the study area. The straight line in the figure represents the fitted model, and the shaded area indicates the confidence interval of the fitted model.

The top-ranked model for detection probability (*p*) identified distance to puddles as the most influential covariate affecting wild boar detectability (Table 1). The detection probability decreased as the distance to the nearest puddle increased (Figure 2). Model-specific β-coefficients from the occupancy models revealed additional covariates significantly affecting detection probability (Table 2). Positive β-coefficients for TRI (β = 0.16), distance to agriculture (β = 2.17), distance to settlement (β = 1.33), NDVI (β = 0.05), and forest types (β = 1.70) suggested that these factors increased the likelihood of wild boar detection. In contrast, negative β-coefficients for distance to road (β = −0.49), distance to river (β = −0.17), and distance to puddle (β = −0.91) indicated that increased distances from these features reduced detection probability.

### 3.3. Factors Affecting Occupancy Probability

**Figure 3:**
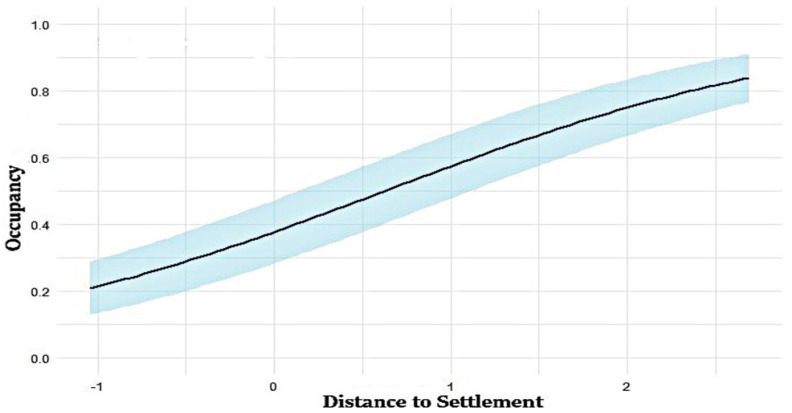
Relationship between distance to settlement and occupancy in the study area. The straight line in the figure represents the fitted model, and the shaded area indicates the confidence interval of the fitted model.

The top-ranked model identified distance to human settlement as the most influential predictor for occupancy probability (ψ), with the model ψ (settlement) *p* (puddle) achieving the highest rank (ΔAIC = 0) (Figure 3). The combination of distance to settlements and distance to puddles provides valuable insights into wild boar occupancy patterns, highlighting the role of human-influenced landscapes in shaping their distribution (Table 3).

## 4. Discussion

Our study provides critical insights into the factors impacting wild boar (*Sus scrofa*) occupancy and detection in the BNP, South Korea. The results indicate that wild boars tend to occupy areas farther from human settlements but closer to water sources such as puddles. This addresses a major research gap in our understanding of wild boar habitat preferences and detection probability in protected areas, particularly in East Asia, where this subject is largely understudied [20,23]. These findings improve our understanding of wild boar distribution and habitat use in protected areas and provide guidance for effective population management and conflict mitigation. 

The distance to settlement was the most influential covariate for wild boar occupancy. The positive association between occupancy probability and distance to settlements (Table 2) suggests that wild boars tend to avoid areas with high levels of human activity, which is consistent with the results of previous studies [33,43]. Studies in Japan, Argentina, Germany, and Spain reported decreased wild boar occupancy near settlements [44,45,46,47], we noted that in certain urban contexts, wild boars exploit human-dominated areas due to food availability and cover. In BNP, Culling and capture were most common near settlement areas, which may have led wild boar to avoid these areas. This indicates that direct human intervention, such as population control strategies, has a major effect on wild boar distribution and habitat selection in human-dominated landscapes. Furthermore, the high levels of noise, artificial lighting, and infrastructure in metropolitan areas could contribute to their avoidance behaviors.

Our findings demonstrate that wild boars preferentially inhabit regions adjacent to agricultural lands, as indicated by the inverse relationship between occupancy probability and proximity to agricultural land (Table 1). This indicates that agricultural lands offer abundant resources, presumably because of the availability of food, including crops and agricultural byproducts [48]. The likelihood of detection increased with distance from agricultural zones despite this preference, likely because of behavioral adaptations to avoid daytime human encounters. Wild boars exhibit considerable adaptability; they often forage nocturnally in agricultural fields and seek refuge in forested or less-disturbed regions during daylight to minimize detection and disturbance [49]. This behavior corresponds with research in other areas where wild boars utilize agricultural land for food while demonstrating cautious movement patterns to reduce human exposure [50,51]. This trade-off between food availability and risk avoidance suggests that although wild boars are drawn to farmland for feeding, they actively avoid the direct presence of humans. Because they are classified as agricultural pests in South Korea, where they inflict considerable crop damage, regulating the movement of these mammals near agricultural areas through deterrents, fencing, and regulated hunting is a vital management priority.

Proximity to water sources, especially puddles, was a critical factor in the detection and occupancy of wild boar in the present study. Our findings demonstrate that wild boar are more likely to occupy and be detected near puddles, as evidenced by the inverse correlation between occupancy and detection probability as the distance from puddles increased. This phenomenon has been observed in other areas, including the United States, where the presence of wild boar declines with increasing distance from water sources [52]. Puddles fulfill vital ecological roles in wild boars by supplying drinking water and enabling wallowing behavior, which is essential for thermoregulation and parasite elimination [45,53]. Nonetheless, their propensity to utilize water sources may increase the risk of disease transmission, particularly that of ASF, which has raised major concerns in South Korea. Thus, monitoring and control initiatives should focus on high-risk zones adjacent to water sources to mitigate disease transmission and restrict population growth.

Wild boars exhibit complex spatial dynamics in response to the presence of roads and rivers. The detection probability had a weak negative relationship with the distance to roads in the present study, suggesting that roads act as partial barriers but do not strongly limit movement. Occupancy probability decreased with distance from the rivers, indicating a preference for areas near the water. However, the weaker negative association between detection probability and distance to rivers suggests that although rivers are important habitat features, their effect on detectability is less pronounced. Additionally, our findings indicate that wild boar are habitat generalists, showing no significant preference for a specific forest type. This aligns with the global observations of their adaptability to various vegetation types [23,54,55]. This habitat flexibility highlights the ability of the species to exploit diverse environments, making it a resilient yet potentially problematic species in human-dominated landscapes.

In South Korea, wild boars are considered pest species because of their impact on agriculture and their likely role in ASF transmission [17,56]. Consequently, government efforts have been directed toward population control rather than conservation. Given the strong influence of settlement avoidance and water availability on wild boar occupancy, future management strategies should prioritize targeted capture or culling efforts, similar to invasive nutria [57], away from human settlements where populations are more established, and near water sources where boars congregate. Additionally, agricultural lands bordering forested regions should be prioritized for population reduction to avoid further crop damage and economic losses. Given the adaptability and resilience of wild boars, a combination of capture, fencing, and public awareness should be implemented to reduce their impact.

Our investigation of wild boar occupancy in the BNP provides valuable insights into the environmental and anthropogenic factors affecting their distribution during spring. However, our study was limited by the small number of cameras (24) and the relatively low detection rate of wild boars, with animals recorded at only 14 locations over the three-month survey period. The short duration of the study may also have limited our ability to capture seasonal variations in occupancy and detection patterns. Therefore, future studies should incorporate a larger number of cameras and long-term monitoring to assess the effect of control measures and the effectiveness of different management strategies in the reduction in wild boar populations.

## 5. Conclusions

Our study provides valuable insights into spring season habitat preferences and detection probabilities of wild boars in urban-proximate protected areas of South Korea. In this study, we identified distance to settlements and proximity to puddles as key determinants of occupancy and detection. These findings contribute to the growing body of knowledge on wild boar ecology in human-dominated landscapes and offer practical guidance for wildlife management. Future research should explore the long-term impact of habitat fragmentation and climate change on wild boar populations, as well as the effectiveness of mitigation measures in reducing human–wildlife conflicts. The integration of ecological research with management actions can ensure the coexistence of wild boar and human communities in shared landscapes.

## Figures and Tables

**Table 1:** Summary of the model-selection procedures for factors influencing the effect of fine-scale covariates on wild boar detection probability.

Model	K	AIC	ΔAIC	AICWt	CumltvWt	Loglikelihood
*p* (Puddle)	3	76.53	0	0.99	0.99	−34.59
*p* (Settlement)	3	87.41	10.88	0.00	0.99	−40.04
*p* (River + Settlement)	4	90.32	13.79	0.00	0.99	−39.98
*p* (Agri)	3	91.83	15.30	0.00	0.99	−42.25
*p* (Null)	2	91.87	15.34	0.00	0.99	−43.62
*p* (Road)	3	92.76	16.23	0.00	0.99	−42.71
*p* (Agri + Road)	4	94.08	17.55	0.00	0.99	−41.86
*p* (TRI)	3	94.27	17.74	0.00	0.99	−43.47
*p* (River)	3	94.44	17.91	0.00	0.99	−43.55
*p* (NDVI)	3	94.53	18.00	0.00	0.99	−43.59
*p* (Road + River)	4	95.07	18.54	0.00	0.99	−42.36
*p* (Forest types)	3	97.23	20.70	0.00	1	−43.44

Note: AIC, Akaike’s information criterion; ΔAIC, difference in AIC values; k, degrees of freedom; CumltvWt, Cumulative Weight. Covariates such as puddle, distance to puddle; settlement, distance to human settlement; agriculture, distance to agricultural land; road, distance to road; tri, terrain ruggedness index; river, distance to river; NDVI, normalized difference vegetation index.

**Table 2:** Model-averaged β-coefficients, lower confidence intervals (LCI), and upper confidence intervals (UCI) for covariates influencing wild boar occupancy and detection probabilities.

	Occupancy			Detection		
Covariates	Β	LCI	UCI	β	LCI	UCI
TRI	−0.22	−1.80	1.35	0.16	−0.43	0.77
Agriculture	−1.09	−1.76	0.14	2.17	0.315	2.93
Settlement	1.71	0.34	3.12	1.33	0.14	2.52
Road	−0.12	−1.97	1.72	−0.49	−0.23	1.22
River	−2.62	−0.893	3.67	−0.17	−1.03	0.68
Puddles	−0.95	−2.31	0.39	−0.91	−1.56	−0.26
NDVI	0.05	−1.38	1.50	0.05	−0.47	0.59
Forest types	1.34	0.25	2.01	1.70	−0.95	4.36

Note: Positive β-coefficients indicate a positive association with occupancy/detection, while negative β-coefficients indicate a negative association. Covariates such as TRI, terrain ruggedness index; and NDVI, normalized difference vegetation index.

**Table 3:** Summary of the model-selection procedures for factors influencing wild boar occupancy.

Model	K	AIC	ΔAIC	AICWt	Loglikelihood	CumltvWt
Psi (Settlement) P (puddle)	4	76.82	0.00	0.25	−33.23	0.25
Psi (River) P (puddle)	4	77.98	1.17	0.14	−33.81	0.39
Psi (Agri) P (puddle)	4	78.20	1.39	0.12	−32.23	0.51
Psi (Road) P (puddle)	4	78.65	1.83	0.10	−34.15	0.61
Psi (Road + River) P (puddle)	5	79.55	2.73	0.06	−34.60	0.68
Psi (TRI) P (puddle)	4	79.55	2.73	0.06	−34.60	0.74
Psi (NDVI) P (puddle)	4	79.55	2.73	0.06	−34.60	0.80
Psi (Forest + Road + Puddle) P (puddle)	7	80.31	3.49	0.04	−29.15	0.85
Psi (TRI + Settlement) P (puddle)	5	80.77	3.96	0.03	−33.51	0.88
Psi (River + Road + TRI) P (puddle)	6	80.79	3.97	0.03	−31.59	0.91
Psi (Puddle + River) P (puddle)	5	81.23	4.41	0.03	−33.74	0.94
Psi (Agri + Road) P (puddle)	5	82.05	5.23	0.02	−34.15	0.96
Psi (Agri + Settlement) P (puddle)	5	82.95	6.13	0.01	−34.60	0.97
Psi (TRI + NDVI) P (puddle)	5	82.95	6.13	0.01	−34.60	0.98
Psi (River + Road + NDVI + TRI) P (puddle)	7	83.61	6.80	0.01	−30.81	0.99
Psi (River + Settlement + Puddle + Road) P (puddle)	7	83.63	6.81	0.01	−30.82	1.00
Psi (.) P (puddle)	2	91.88	15.06	0.00	−43.62	1.00
Psi (Global) P (puddle)	12	114.07	37.25	0.00	−27.70	1.00

## Data Availability

The raw data supporting the conclusions of this article will be made available by the author on request.

## Supplementary Materials

The following supporting information can be downloaded at: https://www.mdpi.com/article/10.3390/ani15233423/s1, Table S1: Number of covariates and their values.
